# Supplementary material for: Resolving the full spectrum of human genome variation using Linked-Reads
Source: Genome Res. 2019 Apr;29(4):635–45. doi: 10.1101/gr.234443.118 (PMC6442396; doi:10.1101/gr.234443.118)
Supplement: Supplemental Material [file supp_gr.234443.118_Supplemental_longranger-2.2.zip › longranger-2.2.tar.bz2!/lr2.2-public/lib/stan/cmdstan/stan/doc/api/html/append__row_8hpp_source.html]

Stan: src/stan/math/matrix/append\_row.hpp Source File


|  |  |
| --- | --- |
|  | Stan  2.5.0  probability, sampling & optimization |


- Main Page
- Related Pages
- Namespaces
- Classes
- Files

- File List
- File Members

All Classes Namespaces Files Functions Variables Typedefs Enumerations Enumerator Friends Macros Pages

- src
- stan
- math
- matrix

append\_row.hpp

Go to the documentation of this file.

1 #ifndef STAN\_\_MATH\_\_MATRIX\_\_APPEND\_\_ROW\_HPP

2 #define STAN\_\_MATH\_\_MATRIX\_\_APPEND\_\_ROW\_HPP

3

4 #include <stan/math/matrix/Eigen.hpp>

5 #include <stan/meta/traits.hpp> //stan::return\_type

6 #include <stan/math/error\_handling/matrix/check\_size\_match.hpp>

7 #include <vector>

8

9 namespace stan {

10  namespace math {

11

12  using Eigen::Dynamic;

13  using Eigen::Matrix;

14  using std::vector;

15  using stan::math::check\_size\_match;

16

17  //matrix append\_row(matrix, matrix)

18  //matrix append\_row(matrix, row\_vector)

19  //matrix append\_row(row\_vector, matrix)

20  //matrix append\_row(row\_vector, row\_vector)

21  template <typename T1, typename T2, int R1, int C1, int R2, int C2>

22  inline Matrix<typename return\_type<T1, T2>::type, Dynamic, Dynamic>

23  append\_row(const Matrix<T1, R1, C1> & A,

24  const Matrix<T2, R2, C2> & B) {

25  int Arows = A.rows();

26  int Brows = B.rows();

27  int Acols = A.cols();

28  int Bcols = B.cols();

29  check\_size\_match("append\_row(%1%)",

30  Acols, "columns of A",

31  Bcols, "columns of B",

32  (double\*)0);

33

34  Matrix<typename return\_type<T1, T2>::type, Dynamic, Dynamic>

35  result(Arows + Brows, Acols);

36  for (int j = 0; j < Acols; j++) {

37  for (int i = 0; i < Arows; i++)

38  result(i, j) = A(i, j);

39  for (int i = Arows, k = 0; k < Brows; i++, k++)

40  result(i, j) = B(k, j);

41  }

42

43  return result;

44  }

45

46  //vector append\_row(vector, vector)

47  template <typename T1, typename T2, int R1, int R2>

48  inline Matrix<typename return\_type<T1, T2>::type, Dynamic, 1>

49  append\_row(const Matrix<T1, R1, 1> & A,

50  const Matrix<T2, R1, 1> & B) {

51  int Asize = A.size();

52  int Bsize = B.size();

53  Matrix<typename return\_type<T1, T2>::type, 1, Dynamic>

54  result(Asize + Bsize);

55  for (int i = 0; i < Asize; i++)

56  result(i) = A(i);

57  for (int i = 0, j = Asize; i < Bsize; i++, j++)

58  result(j) = B(i);

59  return result;

60  }

61

62  //matrix append\_row(matrix, matrix)

63  //matrix append\_row(matrix, row\_vector)

64  //matrix append\_row(row\_vector, matrix)

65  //matrix append\_row(row\_vector, row\_vector)

66  template <typename T, int R1, int C1, int R2, int C2>

67  inline Matrix<T, Dynamic, Dynamic>

68  append\_row(const Matrix<T, R1, C1> & A,

69  const Matrix<T, R2, C2> & B) {

70  check\_size\_match("append\_row(%1%)",

71  A.cols(), "columns of A",

72  B.cols(), "columns of B",

73  (double\*)0);

74

75  Matrix<T, Dynamic, Dynamic>

76  result(A.rows() + B.rows(), A.cols());

77  result << A, B;

78  return result;

79  }

80

81  //vector append\_row(vector, vector)

82  template <typename T, int R1, int R2>

83  inline Matrix<T, Dynamic, 1>

84  append\_row(const Matrix<T, R1, 1> & A,

85  const Matrix<T, R1, 1> & B) {

86  Matrix<T, Dynamic, 1>

87  result(A.size()+B.size());

88  result << A, B;

89  return result;

90  }

91

92  }

93 }

94 #endif

check\_size\_match.hpp

stan::math::check\_size\_match

bool check\_size\_match(const char \*function, T\_size1 i, const char \*name\_i, T\_size2 j, const char \*name\_j, T\_result \*result)

**Definition:** check\_size\_match.hpp:13

traits.hpp

Eigen.hpp

stan::math::append\_row

Matrix< typename return\_type< T1, T2 >::type, Dynamic, Dynamic > append\_row(const Matrix< T1, R1, C1 > &A, const Matrix< T2, R2, C2 > &B)

**Definition:** append\_row.hpp:23


---

[ Stan Home Page ]

*© 2011–2014,
Stan Development Team.*
